# Hamming-shifting graph of genomic short reads: Efficient construction and its application for compression

**DOI:** 10.1371/journal.pcbi.1009229

**Published:** 2021-07-19

**Authors:** Yuansheng Liu, Jinyan Li

**Affiliations:** Data Science Institute, University of Technology Sydney, Sydney, Australia; University of Maryland at College Park, UNITED STATES

## Abstract

Graphs such as de Bruijn graphs and OLC (overlap-layout-consensus) graphs have been widely adopted for the de novo assembly of genomic short reads. This work studies another important problem in the field: how graphs can be used for high-performance compression of the large-scale sequencing data. We present a novel graph definition named Hamming-Shifting graph to address this problem. The definition originates from the technological characteristics of next-generation sequencing machines, aiming to link all pairs of distinct reads that have a small Hamming distance or a small shifting offset or both. We compute multiple lexicographically minimal *k*-mers to index the reads for an efficient search of the weight-lightest edges, and we prove a very high probability of successfully detecting these edges. The resulted graph creates a full mutual reference of the reads to cascade a code-minimized transfer of every child-read for an optimal compression. We conducted compression experiments on the minimum spanning forest of this extremely sparse graph, and achieved a 10 − 30% more file size reduction compared to the best compression results using existing algorithms. As future work, the separation and connectivity degrees of these giant graphs can be used as economical measurements or protocols for quick quality assessment of wet-lab machines, for sufficiency control of genomic library preparation, and for accurate de novo genome assembly.

## Author summary

We present a novel graph-based algorithm to compress next-generation short sequencing reads. The novelty of the algorithm is attributed to a new definition of genomic sequence graph named Hamming-Shifting graph. It consists of edges between distinct reads that have a small Hamming distance or a small shifting offset or both. Efficient construction of Hamming-Shifting graphs is challenging. We introduce a heuristic technique to detect the weight-lightest edges through multiple minimizers from each read, then search the minimum spanning trees and forests of the Hamming-Shifting graph for a high-performance compression of the reads. Our method achieves an additional 10 − 30% file size reduction compared to contemporary compression techniques.

This is a *PLOS Computational Biology* Methods paper.

## Introduction

High-throughput next-generation short-reads sequencing machines have technological characteristics that can be translated into good graph definitions to understand the connectivity of genomic sequences [1, 2]. A primary characteristic is the multi-coverage in-depth sequencing of whole DNA or RNA molecules including on repetitive genome regions, which is prone to producing duplicate reads [3]. We translate this characteristic into a node definition for the graph of genomic reads that: a read having *w* duplicates is defined as a node labeled with the number *w*. The sequencing machines are not perfect, sometimes making minor mistakes in the nucleotide base-calling [4, 5]. Hence, some of these duplicate molecular inserts have actually been read as different sequence strings and all stored in a digital file. We translate this low-rate of sequencing errors into an edge definition that: two reads can be connected if they mismatch only at a few base positions to reflect the fact that the two reads should be the same but they contain tiny sequencing errors. Random fragmentation for DNA or RNA samples in the library preparation is another sequencing characteristic [6]. When combined with the multi-coverage in-depth sequencing characteristic [7], it suggests that many pairs of overlapping or shifting reads can exist. We translate this into another facet of edge definition that: two reads can be connected if a long prefix of one read overlaps with a long suffix of the other [8]. Minor mismatches in the overlaps are also permitted because sequencing errors can happen randomly within and across reads.

Formally, let *RS* = {*r*_1_, *r*_2_, …, *r*_*m*_} be a set of distinct short reads of the same length, and let *d* be a distance threshold between two reads. We define a Hamming-Shifting (HaS) distance graph of *RS* as an undirected graph denoted by *G*_*HaS*_(*d*, *RS*) = (*V*, *E*), where *V* = {*r*_1_, *r*_2_, …, *r*_*m*_} and *E* = {*e* = (*r*_*i*_, *r*_*j*_) | *i* ≠ *j*}, satisfying that the mismatching distance between *r*_*i*_ and *r*_*j*_ is less than or equal to *d*. The *mismatching distance* between *r*_*i*_ and *r*_*j*_ is defined as the Hamming distance between *r*_*i*_ and *r*_*j*_ when they do not shift, or otherwise defined as the number of base mismatches in the overlapping part between *r*_*i*_ and *r*_*j*_, plus the reads’ shifting offset number (non-zero). Each node of the graph is labeled with the number of duplicates of the read in the raw data. Each edge is labeled with the mismatching nucleotide bases between the two reads, and the edge is weighted as the mismatching distance or can be weighted by another distance function of these mismatching bases.

We call *G*_*HaS*_(*d*, *RS*) an HaS-graph of *RS* in short. An HaS-graph of *RS* can be simplified into a Hamming graph of *RS* when only 0-shifting (i.e., offset = 0) edges are allowed in edge construction; or it can be converted into a de Bruijn graph of *RS* if mismatches in the overlapping part of any two reads are not permitted. The definition of *G*_*HaS*_(*d*, *RS*) is close to Coil’s weighted similarity graph of *RS* [9], but the fundamental difference between HaS-graph and Coil’s graph lies in the edge definition. An edge in Coil’s graph measures the frequency of common k-mers between the two reads, but an edge in HaS-graph measures the number of mismatching bases; these two measurements are not convertible.

**Fig 1:**
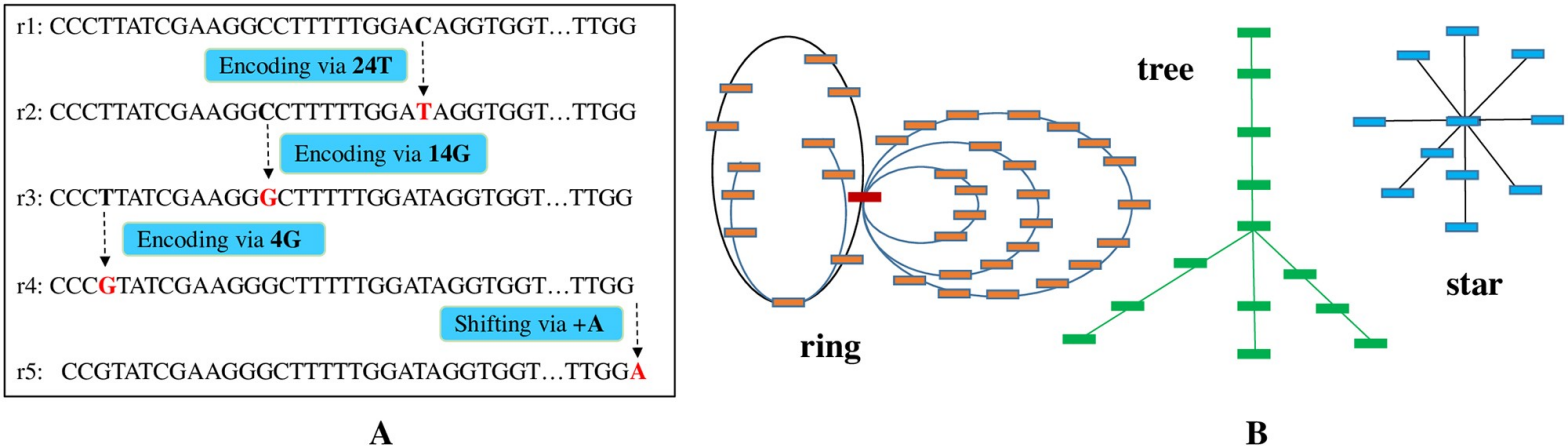
Illustration of mutually referred reads in a path, and various HaS-graph sub-structures. (a) A path containing 5 reads (of the same length 100) for the encoding of r2, r3, r4 and r5. (b) Various types of sub-structures of reads: ring, tree and star.

Due to the above three sequencing characteristics, very often *G*_*HaS*_(*d*, *RS*) is a connected graph for the reads sequenced from one chromosome or from one mRNA molecule. For every path in such a graph, consecutive pairs of reads along the path are all mutually referred via their edge labels. This chained mutual reference in every path of the graph provides an optimal encoding mechanism for high-performance compression of these reads, especially when the path accommodates all of the reads in *RS*. Fig 1A shows an example of *G*_*HaS*_(*d*, *RS*), where *d* = 1 and *RS* = {*r*_1_, *r*_2_, …, *r*_5_}. The first two reads have mismatching bases only at one position (base ‘C’ versus ‘T’ at position 24); while the fourth read has a right-shifting offset 1 with the fifth read, overlapping from the 2nd to 100th position. The first two reads are mutually referred via the edge label ‘24C,T’; the fourth and fifth reads are mutually referred via the edge label ‘+C,A’; and similarly the other two consecutive pairs are also mutually referred.

As a real example, *G*_*HdB*_(*d*, *RS*) for data set SRR445718, where *d* is set as 1, has a long path covering 3742 nodes with 1289 left-shifting edges, 1307 right-shifting edges, and 1146 Hamming edges. Although all consecutive pairs of the nodes have a distance of only 1, the first and the last nodes of the path mismatch at almost all places. It is this chained mutual reference in HaS-graphs that can group *seemingly irrelevant* reads together in one path, signifying a unique advantage over the consensus-based reads-grouping techniques [10, 11] that have a constraint to group only those reads containing a *common* substring.

The compression of the five reads in Fig 1A can start at *r*_2_ using *r*_1_ as reference, with code ‘24T’. This means *r*_2_ can be decoded from *r*_1_ by just replacing *r*_1_’s base at position 24 with ‘T’. Subsequently, *r*_3_ can be encoded using *r*_2_ as reference, with code ‘14G’; *r*_4_ can be encoded using *r*_3_ as reference, with code ‘4G’; *r*_5_ can be encoded using *r*_4_ as reference, with code ‘+A’, which means trimming the head base of *r*_4_ and concatenating base ‘A’ to decode *r*_5_. Therefore, every read (from the second) in a path of *G*_*HaS*_(*d*, *RS*) can be encoded from its immediate previous one via the edge label information. As the reads are chained and mutually referred with minimal codes in the paths, the cascading transfer of the implicit reference leads to very high compression performance—this coding step alone can reduce 100 bytes to 2 bytes for each read in Fig 1A.

In general cases, we search the minimum spanning tree (MST) of *G*_*HaS*_(*d*, *RS*) for the compression of all reads in *RS*. As the weights of the edges in *G*_*HaS*_(*d*, *RS*) are defined as a description function of the mismatching bases between two reads, a minimum spanning tree of *G*_*HaS*_(*d*, *RS*) guarantees minimal total codes for the compression of reads in *RS*. We take a depth-first or breadth-first traversal on the tree to encode all nodes and store all edge labels. In practice for whole genome sequencing, very often *G*_*HaS*_(*d*, *RS*) is not a connected graph. We hence search a minimum spanning forest (MSF) from *G*_*HaS*_(*d*, *RS*), where every connected subgraph induces an MST.

The exhaustive pairwise calculation of the edges’ weight to construct *G*_*HaS*_(*d*, *RS*) is prohibitively expensive because a data set usually contains hundreds of millions of short reads. We apply a heuristic algorithm based on lexicographically sorted substrings to detect a small subset of possibly weight-lightest edges for every read, and then search an MSF from this extremely sparse graph. Prior research [12] has shown that two reads are likely to have a large overlap if they share a *k*-minimizer (the lexicographically smallest *k*-mer). Likewise, large overlap is likely if two reads share a *k*-maximizer (the lexicographically maximal *k*-mer). We exploit the joint power of multiple minimizers and maximizers to index the reads through multiple rounds to detect, with very high probability, the pairs of reads that match at almost every base position.

## Materials and methods

We name our compression method *Mstcom* (short for *m*inimum *s*panning *t*ree based *com*pression for genomic short reads). Mstcom is a lossless compression algorithm for the reads of a fixed length in a FASTQ-format file. It is also applicable to paired-end FASTQ reads of different lengths.

### Overview

Mstcom constructs an MSF from the HaS graph of a set of reads. Every read in the non-root node is encoded using its parent-read as reference in a way similar to Coil [9] or ReCoil [13]. For optimal compression, we set the weight of the edge between *r*_*i*_ and *r*_*j*_ as *w*(*e*(*r*_*i*_, *r*_*j*_)) = *mb* * 3 + *ob*, where *mb* is the number of mismatch bases in the overlapping part of *r*_*i*_ and *r*_*j*_, and *ob* is the number of the offset bases. Analysis about other weight settings are shown in S1 File. The famous Kruskal’s algorithm [14] is used to find the MSF.

**Fig 2:**
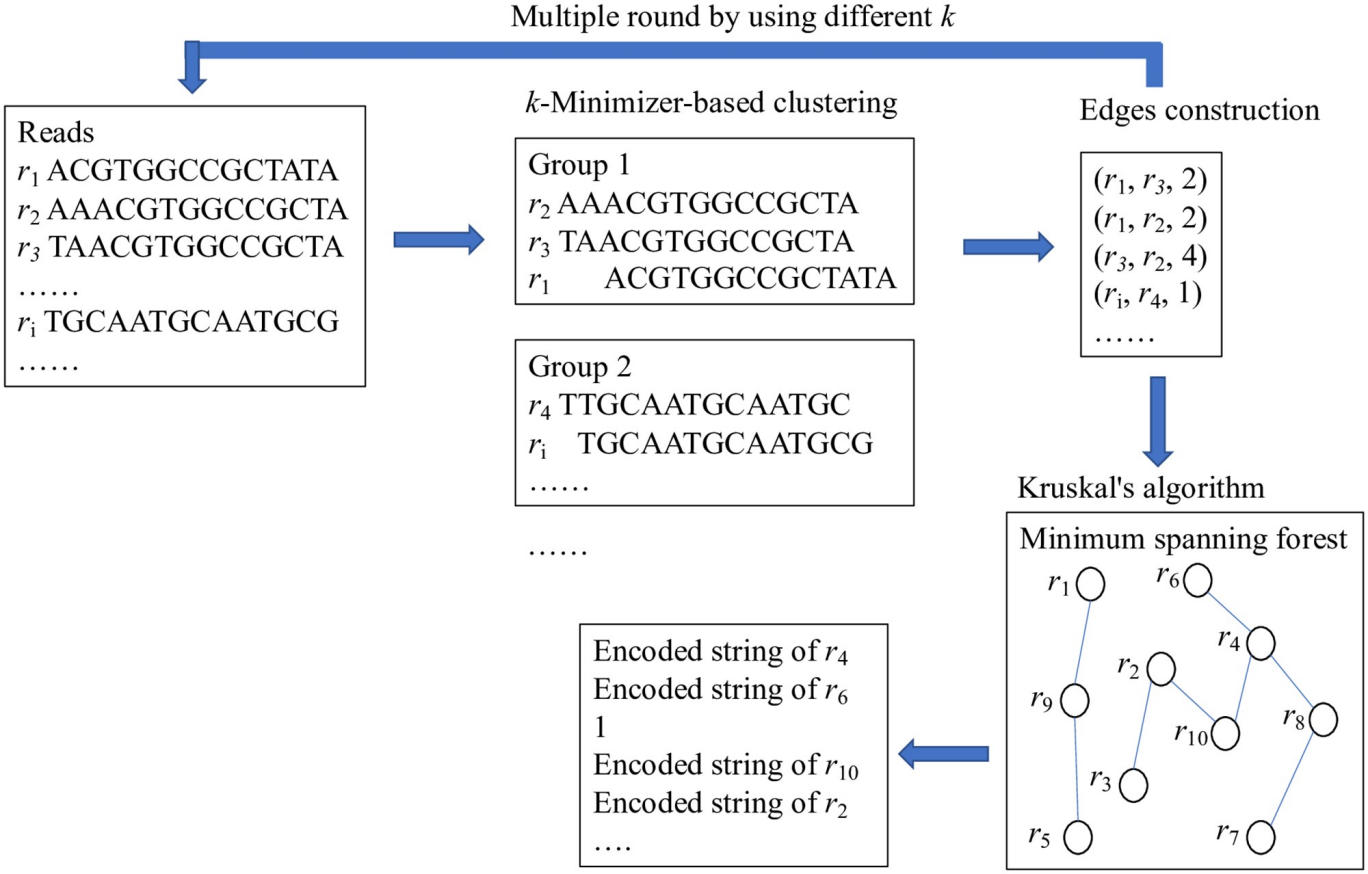
The framework of Mstcom.

The core of the algorithm is the edge construction for the HaS graph. Pairwise comparison of the reads is not practical due to its high computational complexity. Our method indexes reads into blocks via minimizers, where those reads having the same minimizer or maximizer are clustered into the same block. Neighbouring reads are compared in the same block, and then we compute their edge weight using the minimizer as the anchor. The overall workflow of Mstcom is depicted in Fig 2.

### Definitions of minimizers and maximizers

A *k*-minimizer of a string is defined as the smallest *k*-mer under a fixed ordering (e.g., a lexicographic order) in the set of all *k*-mers of the string [12]. An invertible hash function can be used to perform a random ordering of the *k*-mers [15]. The identification of the minimizers is conducted similar to the methods proposed in [11, 16, 17]. We also define the largest *k*-mer of the string as its *k*-maximizer. *k*-maximizers can be independently used to search similar reads.

### Efficient detection of weight-lightest edges

If two reads share the same *k*-minimizer, they will have a common substring of a length at least ≥ *k*. We use these minimizer substrings to index the reads into blocks. Then only those reads in the same block are compared to get their mismatching distance (i.e., their weights) for the construction of the HaS-graph. Here, minimizer is used as the anchor to achieve a fast alignment and comparison. The details of the procedure are as follows:

- **Step 1:** Identify *k*-minimizers for all of the reads and cluster those reads having the same minimizer into the same block;
- **Step 2:** In each block, reads are sorted according to the positions of the minimizer in an ascending order;
- **Step 3:** For each read, search its neighbouring reads in its block to construct its edges. To avoid duplicate edges, it is only compared to the reads ahead of it.

The clustering results, including the number of blocks and the sizes of blocks, are sensitive to the setting of *k*. Assume that two reads have only one pair of mismatching bases, and suppose they are clustered into two different blocks when *k* = *d*. It is the case that they can be clustered into the same block when *k* = *d* − 1. Therefore, some good edges may be missed if only one setting of *k* is used.

To overcome this sensitivity, we use multiple settings of *k* ∈ {*k*_1_, *k*_2_, …, *k*_*n*_} and the above search is carried out across *n* rounds independently. Moreover, *k*-maximizer can be used independently to search the edges as the above search procedure except that *k*-maximizer is identified in **Step 1**. Therefore, we can find the weight-lightest edges through making use of multiple minimizers and maximizers, with a high probability.

For large blocks, the computational complexity involved in **Step 3** can still be high. To handle such cases, we limit the number of reads for the search.

In each round of edge construction (with respect to a specific *k* setting), the edges are sorted by their weight and stored. We note that the search space has slight effects on the compression ratio (as we demonstrate in the S1 File).

We employ parallel computing for the edge construction steps. Specifically in **Step 1**, the identification of each minimizer and/or maximizer is conducted in parallel for each read. Next, the sorting of reads is conducted in parallel for each block. Finally, in **Step 3**, we exploit one last piece of parallelisation. For each read, in parallel, we independently search its neighbouring reads in its block. Then those edges are stored into different files according to their weight. Therefore, the edges can be sorted by their weight after the edge construction step.

### Construction of minimum spanning forests

We employ the well known Kruskal’s algorithm to find the MSF. The steps are:

- **Step 1:** Set each node of the graph as a separate tree;
- **Step 2:** Load edges from the disk in the order of the edge weights from the smallest to largest;
- **Step 3:** For an edge, if it can connect two different trees, i.e., it does not introduce a cycle, then add the edge to the forest and combine the two trees to form one tree.

In implementation, the disjoint-set data structure [18] is used to check whether two nodes are in the same tree or in two different trees.

### Encoding reads on the tree one-by-one through depth-first traversal

Given an MST, any node can be set as its root node. The encoded string of the root node is itself. For every non-root node, it is encoded by using its parent node as reference. The encoded string of a non-root read includes two parts: shifting substring and mismatch information. The shifting substring is followed by the mismatch information. For each mismatch base, the position (delta encoded) and the base are concatenated as a substring. For example, assume that the current read is “ATGCAT”, its parent read is “GCATCC” and the shift offset is 2, then the encoded string of the current read is “AT2G”. An extra bit is used to indicate the shifting direction (left or right direction).

Depth-first search (DFS) is taken to travel the MST for encoding the reads one-by-one. The encoded strings are stored in a text file line-by-line following the DFS node visiting order. When a leaf node is met, a *backtracking step number* is stored in a separate line to tell where the next node to visit is. This information is necessary for recovering the tree structure in decompression. It is easy to distinguish the backtracking step number and the encoded string, since at least a letter is contained in the encoded string but only digital numbers are stored in the line of the backtracking step number.

### Data structures and other details in the encoding

Our method first detects all duplicate reads using 31-minimizer. Those reads sharing the same minimizer at the same position are clustered into the same block. Duplicate reads are detected in each block. As a minimizer is identified from two strands (original strand and its reverse complementary strand), duplicate reads are also distinguished into two types. This duplicate information is stored in the final compressed file.

We use the following five data streams to encode an MSF:

- Stream 1: a text file to store the encoded strings of all nodes (under the DFS order) and the backtracking step numbers (in text numbers).
- Stream 2: a bit stream to tell the shifting direction of an encoded string.
- Stream 3: a bit stream to tell whether a read is encoded on its RC (reverse complementary) strand.
- Stream 4: a bit stream to tell whether a node has duplicate reads;
- Stream 5: if a node has duplicate, two integers are stored. One is for the number of normal duplicate reads and the other is for the number of RC duplicate reads.

All of these data streams are compressed by BSC (http://libbsc.com).

For the compression of paired-end reads, we need to store the pairing information. After vertically concatenating the two FASTQ files, the minimum spanning forest construction follows the same steps as for constructing MSFs from single-end reads. For each read, including duplicate reads, its visiting order is recorded in the DFS traversal. For each pair of reads, the distance in the visiting order after removing the processed reads is stored. In our implementation, the binary indexed tree [19] is used to compute the distance of a pair of reads (see details in the S1 File). An extra bit is used to record the reads order in the pair, i.e., whether the first read of the pair is from the first (_1) file or from the second (_2) file. The distance data stream is compressed by LZMA (http://www.7zip.org).

Mstcom supports compression of paired-end reads having different lengths in the two FASTQ files. Although the reads lengths are different in the two files, the procedure of edge detection and MST construction is not affected. But the relation between reads is more complex and the above encoding step is not adequate. To maintain consistency, in the step of edge detection, we only select those edges having shifting offset when the two reads have different lengths. In the decompression step, the original shifting offset can be recovered from the encoded string as the length of each read and the shifting direction are recorded.

### Order-preserving mode

In the order-preserving mode for compressing single-end reads, the encoded strings are stored in the order of their original reads in the FASTQ file instead of in the DFS visiting order. To recover the tree structure, the ID of the parent read of every non-root node is stored. The ID stream is compressed by BSC. Specially, a duplicating read is stored as an empty line and Streams 4 and 5 are no longer needed. As singleton reads and duplicating reads will never be any parent node, we update the IDs of the reads by removing these two kinds of reads. For example, suppose there are seven reads (*r*_1_, *r*_2_, *r*_3_, *r*_4_, *r*_5_, *r*_6_, *r*_7_), assume *r*_3_ and *r*_6_ are duplicate reads, and assume *r*_2_ is a singleton read. The reads IDs of *r*_1_, *r*_4_, *r*_5_ and *r*_7_ are updated as 1, 2, 3 and 4 respectively.

For compressing paired-end reads under the order-preserving mode, we use the same encoding method as used by the order-free mode for compressing paired-end reads, and an integer stream is used to store the original order of each pair of the reads. This order stream is compressed by BSC.

## Results

The first part of this section presents our theoretical results related to the efficient construction of the best edges for an HaS graph. The theory indicates that our heuristics guarantee a very high probability of producing the globally best edges. In the second part, we present breakthrough compression results and compare them with start-of-the-art compression algorithms. The compression experiments were carried out on a computing cluster running Red Hat Enterprise Linux 7.5 (64 bit) equipped with 2.1 GHz Intel Xeon Platinum 8160 (24 Cores), 384 GB RAM and 5 TB disk space. All algorithms were run with 24 threads under their default/recommended parameters.

### High probability of detecting the weight-lightest edges through multiple rounds of reads indexing

If a read *r* has a large overlap with a read *u*, the read *u* has a high probability to be indexed into *r*’s block. However, it is still possible that there exists a read *v* outside the block that has a shorter distance with *r* than any read *x* inside the block with *r*, namely the weight of edge (*r*, *v*) can be lighter than that of (*r*, *x*). In this case the best (the weight-lightest) edge of *r* is missed for the subsequent compression encoding.

We prove that this probability of missing the best edge is low, and in particular the probability of missing the best edge from *n* blocks is very small, where *n* is the number of different *k* settings (i.e., *k*_1_, *k*_2_, …, *k*_*n*_) for the *k*-minimizer/*k*-maximizer across multiple rounds of indexing.

Our proof is based on a hypothesis used in [15]:

**Hypothesis 1**. Every *k*-mer in a *L*-long read has an equal probability of 1/(*L* − *k* + 1) of being the smallest *k*-mer.

Suppose that the best alignment of two reads has *s* shifting bases. Let *t*_*i*_ represent the number of different *k*-mers in their overlap substring when *k* = *k*_*i*_. Then, the number of *k*-mers in each of the two reads is (*L* − *k*_*i*_ + 1), and the number of common *k*-mers in the overlap substring is (*L* − *k*_*i*_ + 1 − (*s* + *t*_*i*_)).

**Corollary 1**. The probability of the two reads sharing the same *k*_*i*_-minimizer is

$$\begin{array}{c} p\left(k_{i}\right)={\left(\frac{L-k_{i}+1-\left(s+t_{i}\right)}{L-k_{i}+1}\right)}^{2}. \end{array}$$

Let $j=\underset{i}{\arg\max}\left(k_{i}\right)$. For any *k*_*i*_ < *k*_*j*_, the number of different *k*-mers in the overlap substring *t*_*i*_ ≤ *t*_*j*_, and

$$\begin{array}{c} \begin{array}{cc} p\left(k_{i}\right)={\left(\frac{L-k_{i}+1-\left(s+t_{i}\right)}{L-k_{i}+1}\right)}^{2} & >{\left(\frac{L-k_{j}+1-\left(s+t_{i}\right)}{L-k_{j}+1}\right)}^{2}\\ & \geq {\left(\frac{L-k_{j}+1-\left(s+t_{j}\right)}{L-k_{j}+1}\right)}^{2}=p\left(k_{j}\right). \end{array} \end{array}$$

**Proposition 1**. After *n* rounds of independent indexing of the reads based on the *n* minimizers, the probability *p* of two reads being clustered into at least one of the *n* blocks is

$$\begin{array}{c} p=1-\prod_{i=1}^{n}(1-p(k_{i}))>1-{\left(1-p\left(k_{j}\right)\right)}^{n}. \end{array}$$

**Fig 3:**
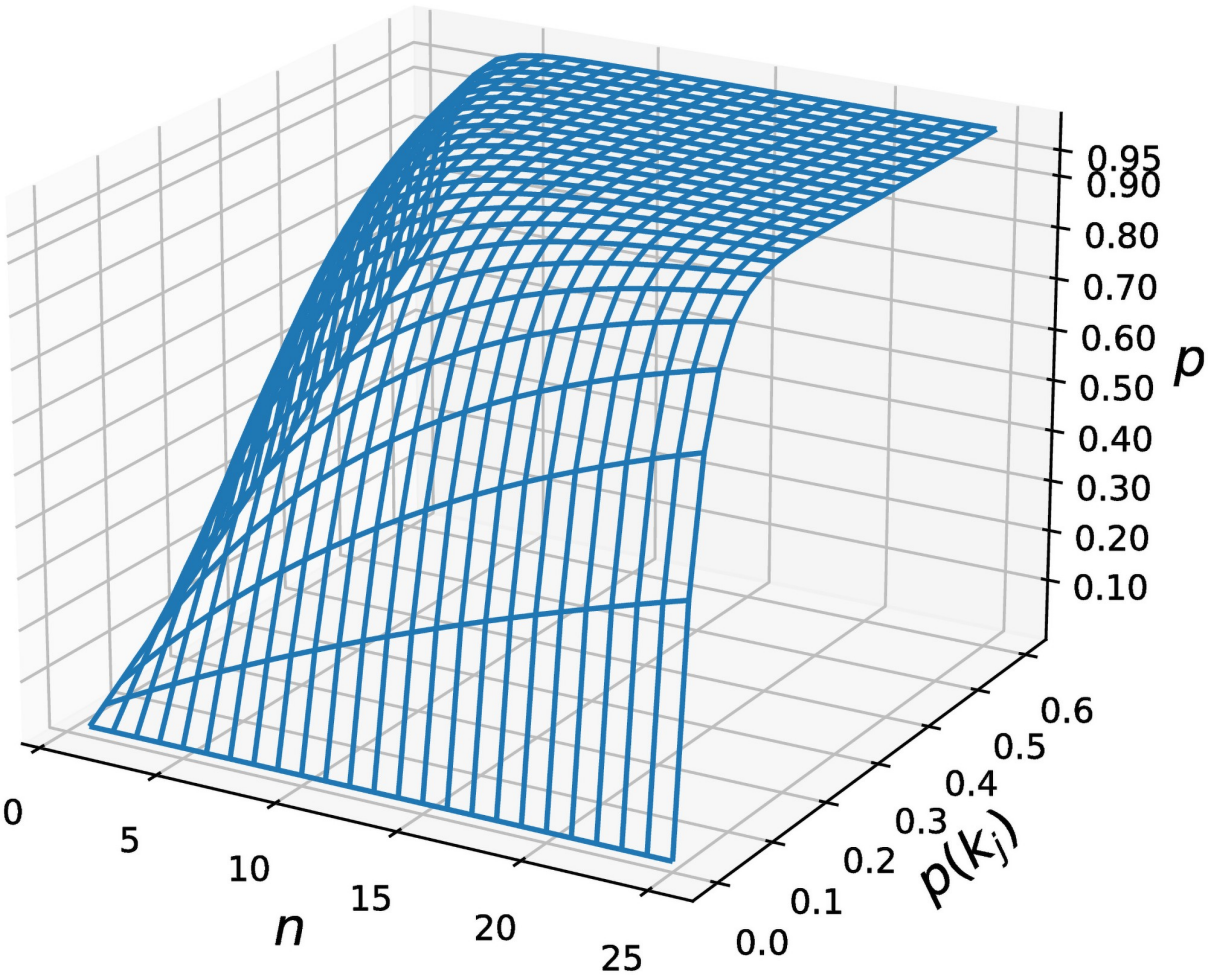
Probabilities of two reads being clustered into one block at lease once in the *n* rounds of indexing. The horizontal lines in the curved grid are scaled at step interval 0.01.

Fig 3 illustrates these probabilities under different settings of *n* and *p*(*k*_*j*_). The probability *p* can be high for a small *p*(*k*_*j*_) when *n* >= 20. For example, when *n* = 20 and *p*(*k*_*j*_) = 0.15, the probability *p* is > 0.961. If *n* = 20 and *p*(*k*_*j*_) = 0.3, *p* will be > 0.999. When *n* = 10 and *p*(*k*_*j*_) = 0.2, *p* > 0.892.

The complexity of this heuristic compared with the global search of the best edges is reduced from *O*(*m*^2^) to $O(\sum_{i=1}^{n}s_{i}^{2})$, where *m* is the number of distinct reads, *n* is the number of blocks, *s*_*i*_ is the size of *i*-th block and $\sum_{i=1}^{n}s_{i}=m$. For a large data set and a small setting of *k* (e.g., 10), a block size can be bigger than one million. The running time is not acceptable in practice. Therefore, we search only a limited scope of reads in such blocks to reduce the complexity further.

### Compression performance on benchmark short-reads data sets

The performance of Mstcom was evaluated on 14 single-end data sets and 7 paired-end data sets, of which 5 are single-cell RNA-seq data sets. These data sets involve a variety of 6 species such as microbial metagenome, *Caenorhabditis elegans*, *Pseudomonas*, *Mus musculus*, *Theobroma cacao* and *Homo sapiens*. There was significant variation in the sequencing depths and the read lengths of these data sets. More details of these data sets are listed in S1 File. The performance was measured under a compression ratio, which is defined as the number of bits per base (bpb) in the compressed file. Mstcom was compared with three of the best compression algorithms published recently: PgRC [20], SPRING [10], and Minicom [11]. Other classical algorithms [21–26] were not compared in detail because they could not outperform Minicom, SPRING, or PgRC.

Our compression performance (under the order-free mode) and performance gain are presented in Table 1. Mstcom outperformed the current methods on all of the benchmark data sets, typically achieving an average improvement of 11% in comparison with the best-performing existing method. Specifically, on the data set SRR870667_1, Mstcom achieved a 27% improvement over the best available result. Compared with SPRING, performance was improved by 22% on average. In particular, an improvement of more than 50% was seen on SRR1265495_1 and SRR870667_1.

**Table 1:** Compression ratios by different methods under the order-free mode.

Type	Data set	Number of bases	SPRING	Minicom	PgRC	Mstcom	Mstcom (speedy)	Gain
SE	ERR174310_1	20, 965, 526, 167	0.4093	0.5716	0.4527	**0.3671**	0.3695	10.30%
	ERR532393_1	3, 575, 287, 300	0.4329	0.4097	0.3765	**0.3175**	0.3223	15.67%
	SRR065389_1	3, 621, 458, 600	0.2238	0.2420	0.1819	**0.1787**	0.1794	1.81%
	SRR1265495_1	1, 699, 645, 675	0.5845	0.3192	0.2894	**0.2513**	0.2535	13.18%
	SRR1294116	4, 643, 407, 633	0.2768	0.2695	0.2415	**0.2143**	0.2184	11.27%
	SRR1313062_1	1, 398, 017, 100	0.4589	0.4951	0.4557	**0.4004**	0.4138	12.13%
	SRR445718	3, 294, 366, 500	0.3404	0.3061	0.2855	**0.2461**	0.2493	13.78%
	SRR445724	5, 089, 652, 800	0.4349	0.3860	0.3444	**0.2918**	0.2953	15.27%
	SRR490961	4, 912, 766, 800	0.2200	0.1996	0.1841	**0.1591**	0.1613	13.54%
	SRR490976	3, 326, 194, 400	0.3919	0.3526	0.3411	**0.2875**	0.2924	15.71%
	SRR554369_1	165, 787, 100	0.2411	0.2881	0.2409	**0.2303**	0.2317	4.43%
	SRR635193_1	1, 472, 357, 574	0.2684	0.2887	0.2599	**0.2189**	0.2233	15.76%
	SRR689233_1	1, 476, 715, 050	0.1947	0.1823	0.1700	**0.1460**	0.1470	14.13%
	SRR870667_1	7, 476, 865, 380	1.2962	0.7348	0.7636	**0.5264**	0.5274	28.36%
PE	ERR174310	41, 931, 052, 334	0.3180	0.4786	0.3071	**0.2948**	0.2963	3.98%
	ERR532393	7, 150, 574, 600	0.5028	0.4714	0.4239	**0.3551**	0.3609	16.23%
	SRR065389	7, 242, 917, 200	0.2375	0.2611	0.1906	**0.1810**	0.1831	5.03%
	SRR1313062	2, 796, 034, 200	0.5641	0.6556	0.5981	**0.5119**	0.5227	9.25%
	SRR554369	331, 574, 200	0.2231	0.2797	0.2207	**0.2177**	0.2194	1.40%
	SRR635193	2, 944, 715, 148	0.3408	0.4187	0.3755	**0.3104**	0.3145	8.91%
	SRR689233	2, 953, 430, 100	0.2410	0.2527	0.2414	**0.1964**	0.1976	18.49%
	ERR174310*	41, 931, 052, 334	0.3647	×	×	**0.3331**	0.3346	10.75%

*Notes*: Mstcom (speedy) stands for Mstcom without the use of maximizers; SE stands for single-end; PE stands for paired-end; Bold font indicates the best compression in the row; ‘*’ means that the paired-end reads have different lengths; ‘×’ indicates that the method cannot compress such a dataset.

The other methods examined have a common step to group similar reads into clusters, and generate a contig within each group de novo as a reference to compress the reads. Our definition for HaS graph and use of MST give rise to the better compression performance because of two key factors. The first is that our method can detect better-quality edges (weight-lighter edges) through the novel use of multiple *k*-minimizers in the multiple rounds of indexing the reads. The second is that every node in a minimum spanning tree allows multiple child nodes. Thus it is not a linear structure, but instead it is a layered structure linking many specific contigs where every branched path can be considered as a contig. Our layered structure is better than PgRC’s linear structure pseudogenome because the encoding of the nodes cannot be globally optimized at a linear structure.

Not only is the compression performance of our Mstcom better than the leading PgRC algorithm, it is also faster on the majority of datasets examined.

In terms of memory usage, Mstcom consumed more memory than all other methods as it had to maintain all of the reads, minimizer-based index structures, and the minimum spanning forest in memory. More details are presented in Table 2 about the running time and memory usage of the four methods, where the total wall-clock time was measured using the */usr/bin/time -v* Unix command.

**Table 2:** Compression time (seconds) and memory usage (GB) of different methods under the order-free mode.

Type	Dataset	SPRING		Minicom		PgRC		Mstcom		Mstcom(speedy)	
		time	memory	time	memory	time	memory	time	memory	time	memory
SE	ERR174310_1	858	10.8	11, 383	80.0	15, 947	19.7	11, 552	65.16	7, 405	64.7
	ERR532393_1	104	4.3	290	9.8	1, 714	5.8	2, 277	18.67	1, 644	16.6
	SRR065389_1	105	3.2	256	8.8	840	2.0	1, 230	18.94	773	16.9
	SRR1265495_1	52	3.3	163	4.9	493	2.1	388	10.88	260	8.4
	SRR1294116	120	3.8	264	9.9	1, 522	4.1	1, 215	19.91	799	17.4
	SRR1313062_1	59	2.5	119	5.7	711	2.6	453	10.26	347	8.8
	SRR445718	112	3.5	232	7.8	1, 378	3.7	1, 124	18.63	640	15.2
	SRR445724	247	4.9	636	12.2	2, 883	8.0	2, 138	28.68	1, 288	23.5
	SRR490961	153	3.8	403	10.3	1, 359	3.5	1, 485	22.09	977	18.8
	SRR490976	149	4.4	274	7.9	1, 810	5.0	1, 309	22.55	865	17.4
	SRR554369_1	4	0.7	20	1.7	31	0.2	39	2.73	25	1.5
	SRR635193_1	52	2.2	170	5.3	435	1.7	444	10.29	298	8.7
	SRR689233_1	42	2.9	76	4.2	311	0.8	373	9.55	236	7.9
	SRR870667_1	422	8.9	744	19.3	15, 410	18.6	5, 074	38.64	3, 132	36.7
PE	ERR174310	1, 452	20.9	20, 895	120.0	20, 313	35.4	24, 953	112.94	15, 774	113.0
	ERR532393	218	5.8	572	17.9	3, 461	9.5	5, 302	30.10	3, 586	27.2
	SRR065389	191	4.1	422	15.2	1, 263	3.4	2, 304	28.80	1, 729	25.7
	SRR1313062	128	2.9	218	9.6	1, 389	4.8	920	17.83	744	15.8
	SRR554369	8	0.8	24	2.0	52	0.3	72	6.51	51	3.4
	SRR635193	111	2.8	317	9.0	793	2.7	753	18.17	627	15.4
	SRR689233	86	3.6	144	7.1	624	1.4	756	15.97	512	13.7
	ERR174310*	1, 921	20.9	−	−	−	−	35, 357	113.08	21, 641	113.15

*Notes*: SE stands for single-end; PE stands for paired-end.

Computational resources used in decompression are presented in Table 3, showing little difference in the running time or the memory usage across the methods.

**Table 3:** Decompression time (seconds) and memory usage (GB) by different methods under the order-free mode.

Type	Dataset	SPRING		Minicom		PgRC		Mstcom	
		time	memory	time	memory	time	memory	time	memory
SE	ERR174310_1	85	6.0	61	4.1	134	9.4	673	32.0
	ERR532393_1	18	3.5	15	0.6	19	1.2	99	4.7
	SRR065389_1	17	1.9	14	0.6	11	0.6	73	5.1
	SRR1265495_1	10	2.7	5	0.2	6	0.5	38	1.6
	SRR1294116	20	3.0	27	0.6	18	1.2	93	4.0
	SRR1313062_1	9	1.9	17	0.3	8	0.6	46	1.6
	SRR445718	17	2.7	17	0.6	15	1.1	75	3.3
	SRR445724	23	4.2	28	1.3	27	2.2	137	5.0
	SRR490961	20	2.8	30	0.9	17	1.2	83	4.3
	SRR490976	17	2.8	38	0.6	19	1.3	84	3.5
	SRR554369_1	1	0.4	4	0.1	6	0.1	4	0.2
	SRR635193_1	10	1.4	15	0.3	5	0.4	29	1.3
	SRR689233_1	8	1.4	3	0.3	8	0.3	24	1.5
	SRR870667_1	43	5.4	37	6.3	88	12.6	312	13.0
PE	ERR174310	177	6.4	210	23.2	272	13.6	2, 367	118.9
	ERR532393	41	5.5	129	4.0	73	2.5	333	19.1
	SRR065389	37	2.9	34	4.1	29	1.8	221	19.4
	SRR1313062	22	2.7	22	1.9	31	1.5	310	8.0
	SRR554369	2	0.6	1	0.2	1	0.1	14	0.9
	SRR635193	21	2.1	58	1.8	21	1.3	210	7.2
	SRR689233	15	2.3	20	1.8	16	0.8	107	6.8
	ERR174310*	286	4.7	−	−	−	−	2, 470	118.9

*Notes*: SE stands for single-end; PE stands for paired-end.

Reducing the number of *k* settings is an easy way to reduce the running time of Mstcom. We tested the performance of Mstcom without using any maximizers (the method is named Mstcom(speedy)). The results are shown in the second-last column of Tables 1 and 2. We can see that a minor sacrifice in compression performance gives rise to dramatic speed increasing, reducing overall time taken by approximately 50% and generating performance that is much faster than PgRC across most cases. A question for future work is how to choose an optimal panel of *k* settings to optimally balance run times and compression performance for Mstcom. Some ideas from SPRING or Minicom could be adopted for this purpose as they are currently faster than Mstcom.

Compression performance under the order-preserving mode are presented in S1 File. Mstcom outperforms all the state-of-the-art methods in terms of compression ratio on 18 data sets. For the remaining 3 data sets, PgRC provides slightly better compression.

### Impact of different numbers of *k*-minimizers and *k*-maximizers on the compression ratio

In our implementation, we set *k*_1_ as the maximum value of *k*_*i*_ and then set *k*_*i*_ = *k*_*i*−1_ − 1 for *i* > 1.

**Fig 4:**
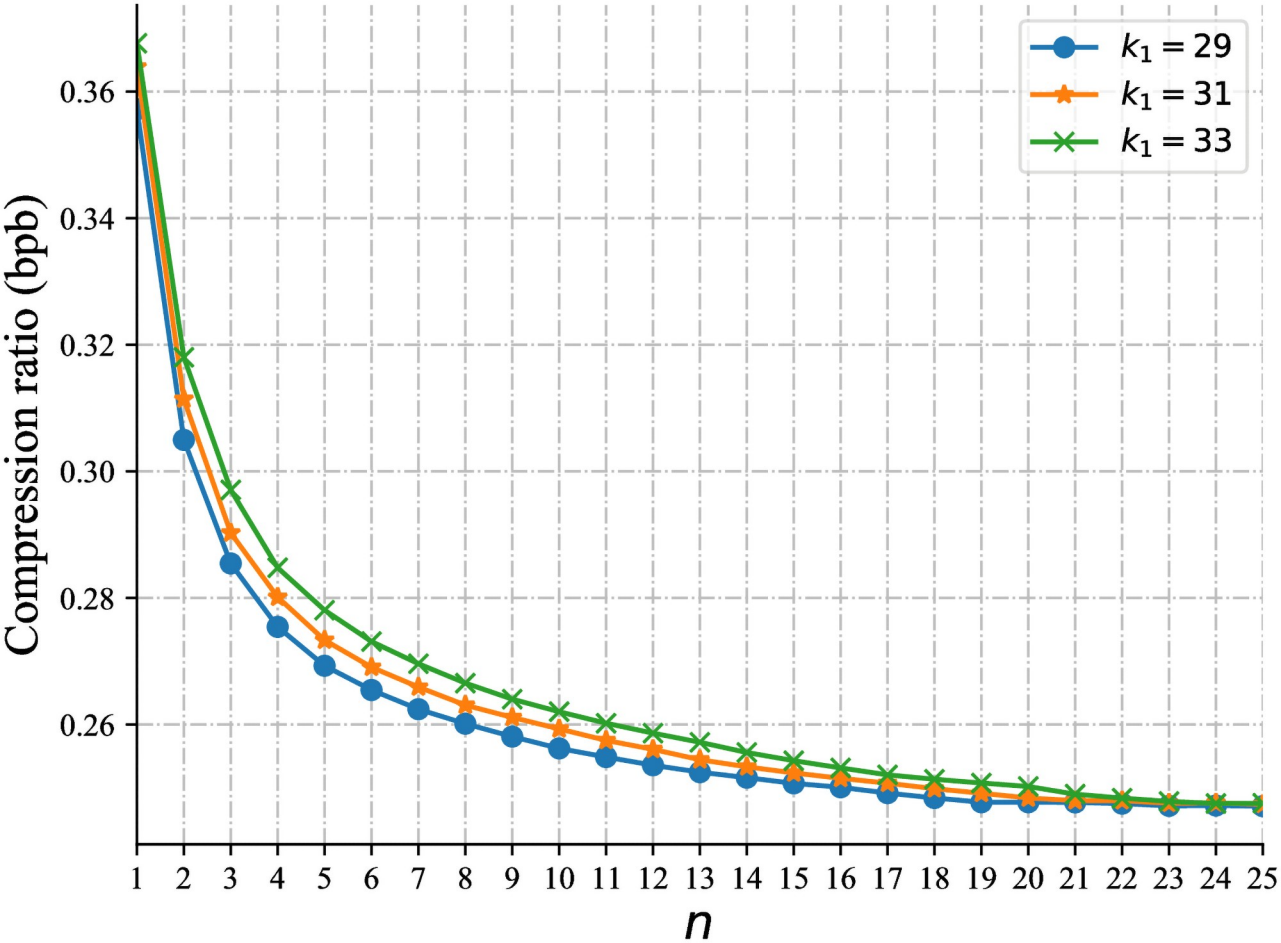
Compression ratios under different settings of *k*_1_ and *n*.

Our reported findings are based on the performance when *n* = 20 and *k*_1_ = 29. To understand the impact of varying *n* and *k*_1_, we examined compression performance on SRR445719 under different *k*_1_ and *n* setting (see Fig 4). The highest compression ratio is achieved when *n* ≥ 20. When *n* is small, e.g., *n* = 1 or 5, the compression ratio is much worse. These results verified our probability analysis on the use of multiple minimizers for edge construction.

## Discussion

Various types of frequent subgraphs in *G*_*HaS*_(*d*, *RS*) are interesting, including ring structures, tree-structures, linear chains, wheels, or star structures (see schematic examples in Fig 1B). These subgraphs are biologically meaningful. For example, a star structure means that the middle read is prone to sequencing errors or random fragmentation. We have studied separation degrees of some HaS graphs. It was expected that for a whole genome sequencing reads set *RW*, *G*_*HaS*_(*d*, *RW*) should be a large connected graph even when the distance threshold *d* is set as a small number such as 2 or 3, or it should contain large connected subgraphs. For a messenger RNA sequencing reads set *RE*, *G*_*HaS*_(*d*, *RE*) should be a disconnected graph, where the reads from the same gene should be connected as a subgraph separated from other subgraphs. However, for the whole genome sequencing data set ERR174310, there exists only one big connected subgraph containing 22, 756, 808 reads when *d* = 5; the other 41 million subgraphs each contain only two reads. This discrepancy implies that: (i) there would have had faulty manipulation on the raw data before publication; (ii) the sequencing machine had a poor distribution of sequencing depths or did not have ideal random fragmentation of the DNA or RNA samples; or (iii) both. Therefore, separation and connectivity degrees of these giant graphs of reads can be used as economical measurements or protocols for a quick quality assessment of the wet-lab machines, for a sufficiency control of genomic library preparation, or for a fast flaw detection of database management issues. Exploring the proposed graph representation for quality control of sequencing data will be explored in our future work.

Our encoding algorithm can be effectively adapted for high-performance compression of human chromosome or genome databases. The first step is to convert every genome into a feature vector, followed by conducting unsupervised clustering on these vectors to group the genomes, and then detecting maximal exact matches or maximal exact matches containing mutations. The mutual reference between the chromosomes or genomes is bridged by the sets of maximal exact matches. If every chromosome or genome is denoted by a node in a graph, the MST of such a graph provides an optimal mechanism to encode all of the sequences for compression.

## Conclusion

In the understanding of connectivities of genomic short reads, we found that edge definition is important for organizing these reads as a graph. In our work, we have proposed to use Hamming distance and shifting offsets to define the label and weight of the edges. Our HaS graphs and the depth-first traversal encoding on the minimum spanning trees have shown great potential in the compression of genomic short reads. The compression performance can be 10 − 30% better than the current state-of-the-art algorithms. Another important contribution made by this work is in the efficient detection of the weight-lightest edges for HaS graphs. We have proposed to use multiple minimizers and maximizers to index the reads, to limit the search scope of the best edges. It has also been proven that this search strategy can reach the solution with a high probability.

Using HaS graphs of genomic reads as a platform for de novo genome assembly is a highly promising future research direction. The edge information (labels and weights) and the copy numbers labeled in the nodes are key information for comprehending the real sequencing coverage and depths at different regions of the genome, and they are also useful for detecting repetition regions. In particular, the quality of the assembled genome would be further improved when the graph is combined with single-cell multi-omics long reads sequencing data.

## Supporting information

S1 File Supplementary document. Details about data sets, results of different methods under order-preserving mode and some parameter analysis of Mstcom.
(PDF)
