# Supplementary material for: Hamming-shifting graph of genomic short reads: Efficient construction and its application for compression
Source: PLoS Comput Biol. 2021 Jul 19;17(7):e1009229. doi: 10.1371/journal.pcbi.1009229 (PMC8321399; doi:10.1371/journal.pcbi.1009229)
Supplement: S1 File — Details about data sets, results of different methods under order-preserving mode and some parameter analysis of Mstcom. (PDF) [file pcbi.1009229.s001.pdf]

Supplementary Material "Hamming-Shifting graph of genomic short reads: efficient construction and its application for compression"

Yuansheng Liu[1] and Jinyan Li[1]

[1]Data Science Institute, University of Technology Sydney, Ultimo, 2007 NSW, Australia

July 9, 2021

1 Data set information

ERR174310 (Number of reads pairs: 207,579,467; Reads length: 101)
ftp://ftp.sra.ebi.ac.uk/vol1/fastq/ERR174/ERR174310/ERR174310_1.fastq.gz
ftp://ftp.sra.ebi.ac.uk/vol1/fastq/ERR174/ERR174310/ERR174310_2.fastq.gz

ERR174310* (Number of reads pairs: 207,579,467; Reads length: 100 and 102)
https://www.ncbi.nlm.nih.gov/sra/?term=ERR174310
Download it by using the following two commands:
1. `wget https://sra-pub-run-odp.s3.amazonaws.com/sra/ERR174310/ERR174310`
2. `fastq-dump --split-files ./ERR174310`

ERR532393 (Number of reads pairs: 35,752,873; Reads length: 100)
ftp://ftp.sra.ebi.ac.uk/vol1/fastq/ERR532/ERR532393/ERR532393_1.fastq.gz
ftp://ftp.sra.ebi.ac.uk/vol1/fastq/ERR532/ERR532393/ERR532393_2.fastq.gz

SRR065389 (Number of reads pairs: 36,214,586; Reads length: 100)
ftp://ftp.sra.ebi.ac.uk/vol1/fastq/SRR065/SRR065389/SRR065389_1.fastq.gz
ftp://ftp.sra.ebi.ac.uk/vol1/fastq/SRR065/SRR065389/SRR065389_2.fastq.gz

SRR1265495_1 (Number of reads: 16,828,175; Reads length: 101)
ftp://ftp.sra.ebi.ac.uk/vol1/fastq/SRR126/005/SRR1265495/SRR1265495_1.fastq.gz

SRR1294116 (Number of reads: 45,974,333; Reads length: 101)
ftp://ftp.sra.ebi.ac.uk/vol1/fastq/SRR129/006/SRR1294116/SRR1294116.fastq.gz

SRR1313062 (Number of reads pairs: 27,960,342; Reads length: 50)
ftp://ftp.sra.ebi.ac.uk/vol1/fastq/SRR131/002/SRR1313062/SRR1313062_1.fastq.gz
ftp://ftp.sra.ebi.ac.uk/vol1/fastq/SRR131/002/SRR1313062/SRR1313062_2.fastq.gz

SRR445718 (Number of reads: 32,943,665; Reads length: 100)
ftp://ftp.sra.ebi.ac.uk/vol1/fastq/SRR445/SRR445718/SRR445718.fastq.gz

SRR445724 (Number of reads: 50,896,528; Reads length: 100)
ftp://ftp.sra.ebi.ac.uk/vol1/fastq/SRR445/SRR445724/SRR445724.fastq.gz

SRR490961 (Number of reads: 49,127,668; Reads length: 100)
ftp://ftp.sra.ebi.ac.uk/vol1/fastq/SRR490/SRR490961/SRR490961.fastq.gz

SRR490976 (Number of reads: 33,261,944; Reads length: 100)
ftp://ftp.sra.ebi.ac.uk/vol1/fastq/SRR490/SRR490976/SRR490976.fastq.gz

SRR554369 (Number of reads pairs: 1,657,871; Reads length: 100)

ftp://ftp.sra.ebi.ac.uk/vol1/fastq/SRR554/SRR554369/SRR554369_1.fastq.gz
ftp://ftp.sra.ebi.ac.uk/vol1/fastq/SRR554/SRR554369/SRR554369_2.fastq.gz

SRR635193 (Number of reads pairs: 27,265,881; Reads length: 54)
ftp://ftp.sra.ebi.ac.uk/vol1/fastq/SRR635/SRR635193/SRR635193_1.fastq.gz
ftp://ftp.sra.ebi.ac.uk/vol1/fastq/SRR635/SRR635193/SRR635193_2.fastq.gz

SRR689233 (Number of reads pairs: 16,407,945; Reads length: 90)
ftp://ftp.sra.ebi.ac.uk/vol1/fastq/SRR689/SRR689233/SRR689233_1.fastq.gz
ftp://ftp.sra.ebi.ac.uk/vol1/fastq/SRR689/SRR689233/SRR689233_2.fastq.gz

SRR870667_1 (Number of reads: 69,230,235; Reads length: 108)
ftp://ftp.sra.ebi.ac.uk/vol1/fastq/SRR870/SRR870667/SRR870667_1.fastq.gz

Table A: Details of datasets

Dataset	Layout	Coverage	Instrument Model	Library Strategy	Species
ERR174310	Paired	14.0	Illumina HiSeq 2000	WGS	*Homo sapiens*
ERR532393	Paired	–	Illumina HiSeq 2000	WGS	Metagenome
SRR065389	Paired	65.1	Illumina Genome Analyzer II	WGS	*Caenorhabditis elegans*
SRR1265495	Single	1.3	Illumina HiSeq 2000	RNA-Seq	*Homo sapiens*
SRR1294116	Single	1.3	Illumina HiSeq 2000	RNA-Seq	*Homo sapiens*
SRR1313062	Paired	0.6	Illumina HiSeq 2000	RNA-Seq	*Homo sapiens*
SRR445718*	Single	0.9	Illumina HiSeq 2000	RNA-Seq	*Homo sapiens*
SRR445724*	Single	1.2	Illumina HiSeq 2000	RNA-Seq	*Homo sapiens*
SRR490961*	Single	1.3	Illumina HiSeq 2000	RNA-Seq	*Homo sapiens*
SRR490976*	Single	0.8	Illumina HiSeq 2000	RNA-Seq	*Homo sapiens*
SRR554369	Paired	1.8	Illumina Genome Analyzer IIx	WGS	*Pseudomonas*
SRR635193	Paired	0.7	Illumina Genome Analyzer IIx	RNA-Seq	*Homo sapiens*
SRR689233*	Paired	1.1	Illumina HiSeq 2000	RNA-Seq	*Mus musculus*
SRR870667	Paired	21.2	Illumina Genome Analyzer IIx	WGS	*Theobroma cacao*

Note: * indicates single cell RNA-seq data.

2 Performance of order-preserving

The compression ratio, running time and memory usage are presented in Figures B, C and D.

3 Search space setting

Let u be the number of searched reads in each group and v be the number of edges stored for each reads in a round. In our implementation, in each round, for each reads, we search its previous reads in its group. Here, we only search at most u number of reads. Then sort these edges by their weight and storing v number of edges having smallest weight. Figure A presents compression ratios under different u and v on the data set SRR445718. The search space and the number of stored edges has little effect (< 0.001) on the compression ratio. Note that search more reads and store more edges spend more time. Considering the running time and disk space, we set $u = 400$ and $v = 10$.

4 Edge weight setting

In our method, we set $f(s, m) = s + t \cdot m$. Figure B depicts the compression ratio on three data sets by using different t. Finally, we set $t = 3$.

In addition, we find that the weight threshold d has little effect on the compression if d is not too small. Therefore, we set $d = L - 10$, where L is the length of reads.

Table B: Compression ratios - order-preserving

Type	Dataset	SPRING	Minicom	PgRC	Mstcom	Gain
SE	ERR174310_1	0.6966	0.8425	0.7236	**0.6445**	7.47%
	ERR532393_1	0.6668	0.6465	0.6206	**0.5619**	9.46%
	SRR065389_1	0.4784	0.4926	**0.4235**	0.4242	−0.17%
	SRR1265495_1	0.6979	0.4482	**0.3767**	0.3917	−3.99%
	SRR1294116	0.5131	0.5022	0.4829	**0.4479**	7.25%
	SRR1313062_1	0.9034	0.9193	0.9236	**0.8375**	7.30%
	SRR445718	0.5746	0.5355	0.5346	**0.4831**	9.64%
	SRR445724	0.6622	0.6073	0.5984	**0.532**	11.09%
	SRR490961	0.4605	0.4398	0.4336	**0.4003**	7.67%
	SRR490976	0.6057	0.5664	0.5879	**0.5249**	7.31%
	SRR554369_1	0.4432	0.4907	0.4385	**0.4309**	1.73%
	SRR635193_1	0.6982	0.7022	0.6842	**0.6222**	9.06%
	SRR689233_1	0.4492	0.4374	0.4303	**0.3966**	7.84%
	SRR870667_1	1.4587	0.9701	1.0332	**0.7766**	19.95%
PE	ERR174310	0.4640	−	0.4395	**0.4382**	0.30%
	ERR532393	0.6211	−	0.5381	**0.4875**	9.40%
	SRR065389	0.3631	−	**0.3039**	0.3098	−1.96%
	SRR1313062	0.7986	−	0.7991	**0.7708**	3.48%
	SRR554369	0.3234	−	**0.3133**	0.3187	−1.74%
	SRR635193	0.5646	−	0.5444	**0.5425**	0.35%
	SRR689233	0.3688	−	0.3566	**0.3263**	8.50%

Note: Bold font indicates the best result in the row.

Table C: Compression time (seconds) and memory (GB) - order-preserving

Type	Dataset	SPRING		Minicom		PgRC		Mstcom	
		time	memory	time	memory	time	memory	time	memory
SE	ERR174310_1	867	10.8	11,383	80.0	18,153	23.4	7,554	65.0
	ERR532393_1	105	3.4	290	9.8	1,784	6.0	1,954	16.2
	SRR065389_1	106	3.2	290	8.8	830	2.4	734	17.0
	SRR1265495_1	49	2.8	110	4.9	502	2.3	257	8.5
	SRR1294116	125	3.3	290	9.9	1,573	4.5	725	17.4
	SRR1313062_1	63	2.3	110	5.7	728	2.8	346	8.8
	SRR445718	118	3.3	230	7.8	1,385	4.0	704	14.8
	SRR445724	245	3.3	650	12.2	2,968	8.4	1,393	23.6
	SRR490961	165	3.3	410	10.3	1,414	3.9	963	18.9
	SRR490976	155	3.3	290	7.9	1,743	5.2	867	17.4
	SRR554369_1	4	0.7	50	1.7	31	0.2	24	1.7
	SRR635193_1	55	2.2	170	5.3	463	1.9	303	8.8
	SRR689233_1	40	2.9	110	4.2	319	1.1	239	7.9
	SRR870667_1	420	7.7	770	19.3	16,043	19.1	3,215	36.9
PE	ERR174310	1,419	20.9	20,895	120.0	23,283	37.0	15,634	113.1
	ERR532393	220	4.4	572	17.9	3,389	9.8	4,057	27.4
	SRR065389	191	4.1	422	15.2	1,396	3.7	1,745	26.0
	SRR1313062	123	2.9	218	9.6	1,427	5.0	741	16.1
	SRR554369	7	0.8	24	2.0	52	0.3	51	3.4
	SRR635193	113	2.8	317	9.0	840	2.9	622	15.4
	SRR689233	83	3.6	144	7.1	643	1.6	513	13.9

Table D: Decompression time (seconds) and memory (GB) - order-preserving

Type	Dataset	SPRING		Minicom		PgRC		Mstcom	
		time	memory	time	memory	time	memory	time	memory
SE	ERR174310_1	115	4.7	183	23.2	226	10.8	1047	32.6
	ERR532393_1	19	1.9	41	4.0	29	1.3	154	5.5
	SRR065389_1	16	1.9	38	4.1	25	0.7	139	5.5
	SRR1265495_1	8	2.0	19	1.9	8	0.5	50	2.6
	SRR1294116	32	1.9	47	5.1	27	1.3	161	7
	SRR1313062_1	13	1.4	26	1.9	13	0.7	83	2.9
	SRR445718	17	1.8	39	3.7	21	1.2	132	5
	SRR445724	25	2.3	52	5.7	37	2.3	203	7.9
	SRR490961	22	1.8	49	5.5	26	1.3	156	7.4
	SRR490976	20	2.3	40	3.7	24	1.4	140	5.1
	SRR554369_1	5	0.3	1	0.2	6	0.1	8	0.3
	SRR635193_1	11	1.4	26	1.8	10	0.5	68	2.8
	SRR689233_1	8	1.4	11	1.8	7	0.3	53	2.5
	SRR870667_1	52	3.9	69	8.8	117	13.1	399	12.1
PE	ERR174310	215	5.5	–	–	510	13.8	3065	118.9
	ERR532393	55	3.5	–	–	93	2.4	394	19.1
	SRR065389	37	2.9	–	–	54	1.2	275	19.4
	SRR1313062	26	2.2	–	–	39	1.2	314	8
	SRR554369	8	0.6	–	–	1	0.1	15	0.9
	SRR635193	23	2.1	–	–	33	0.8	247	7.2
	SRR689233	22	2.3	–	–	21	0.6	130	6.8

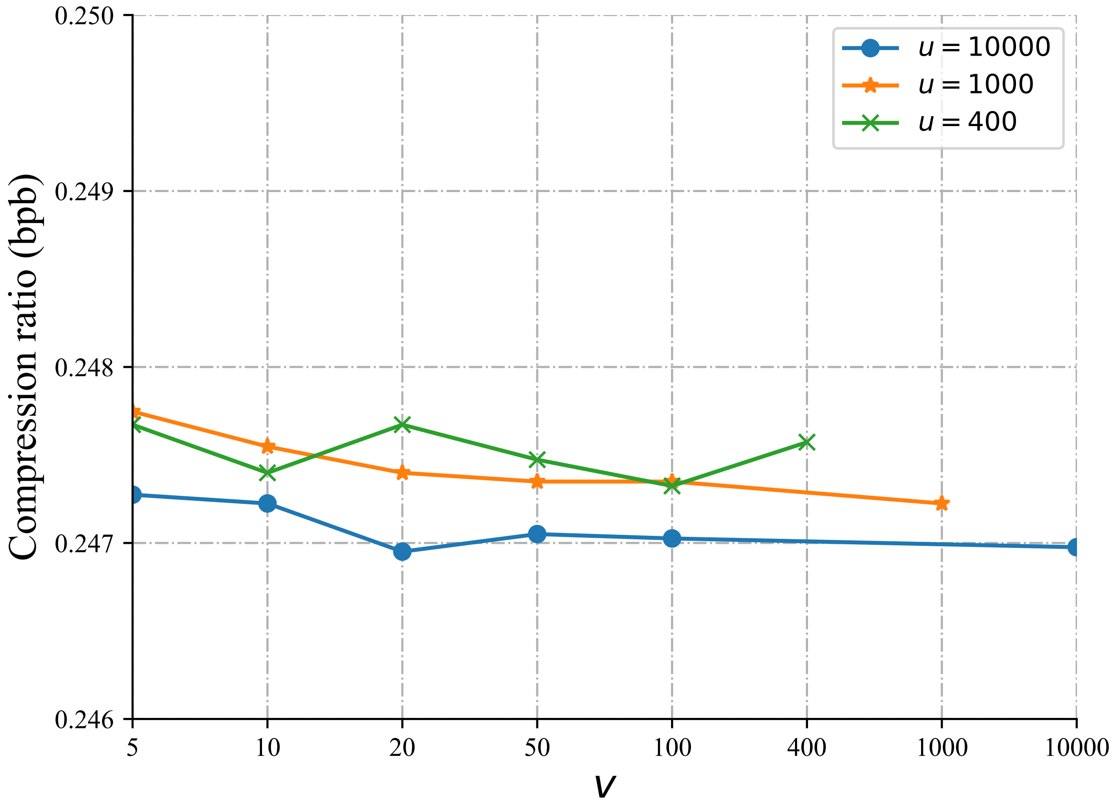

Figure A: Compression ratios under different u and v.

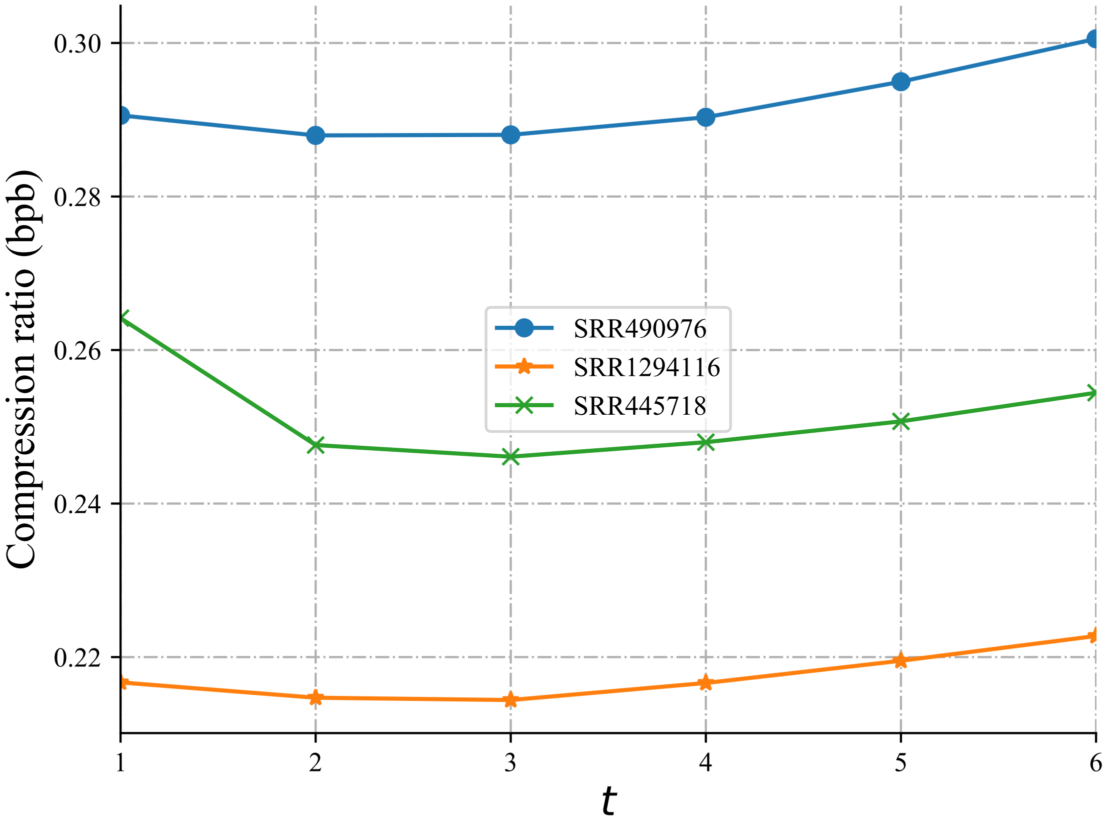

Figure B: Compression ratios on three data sets using different t.

5 Compression of paired-end reads

The pairing information is stored as the distance of pair of reads. For example, four reads (r_1, r_2, r_3, r_4) are in "_1" file and four reads (r_5, r_6, r_7, r_8) are in "_2" file. The compression is the same as compression of single-end reads $(r_1, r_2, \cdots, r_7, r_8)$. Assume that the encoding order is $r_4, r_2, r_1, r_7, r_8, r_3, r_6, r_5$. For the read r_4, its pairing read is r_8 and the distance between r_4 and r_8 is 3. In addition, a bit is used to label the order of this pair reads. Next, for the read r_2, its pairing read is r_6 and the distance between r_2 and r_6 is $(4 - 1) = 3$ as r_8 is processed read. That is to say, the distance is the number of reads unprocessed between current pair reads. It is calculated by using the number of reads between the pair reads minus the number of processed pair reads. In implementation, the binary indexed tree is used to count the number of processed pair reads.

6 Compared tools and exact commands used in experiment

SPRING (version from 2020-July-1; https://github.com/shubhamchandak94/Spring)

Minicom (version from 2020-July-1; https://github.com/yuansliu/minicom)

PgRC (v1.1, clone at 2019-November-30; https://github.com/kowallus/PgRC)

Mstcom (https://github.com/yuansliu/mstcom)

Exact commands of the four methods are reported in the page https://github.com/yuansliu/mstcom/tree/master/script.
